# Recovery of protein synthesis to assay DNA repair activity in transcribed genes in living cells and tissues

**DOI:** 10.1093/nar/gkad642

**Published:** 2023-07-31

**Authors:** Melanie van der Woude, Carlota Davó-Martínez, Karen L Thijssen, Wim Vermeulen, Hannes Lans

**Affiliations:** Department of Molecular Genetics, Erasmus MC Cancer Institute, Erasmus University Medical Center, 3015 GD, Rotterdam, The Netherlands; Department of Molecular Genetics, Erasmus MC Cancer Institute, Erasmus University Medical Center, 3015 GD, Rotterdam, The Netherlands; Department of Molecular Genetics, Erasmus MC Cancer Institute, Erasmus University Medical Center, 3015 GD, Rotterdam, The Netherlands; Department of Molecular Genetics, Erasmus MC Cancer Institute, Erasmus University Medical Center, 3015 GD, Rotterdam, The Netherlands; Department of Molecular Genetics, Erasmus MC Cancer Institute, Erasmus University Medical Center, 3015 GD, Rotterdam, The Netherlands

## Abstract

Transcription-coupled nucleotide excision repair (TC-NER) is an important DNA repair mechanism that protects against the negative effects of transcription-blocking DNA lesions. Hereditary TC-NER deficiencies cause pleiotropic and often severe neurodegenerative and progeroid symptoms. While multiple assays have been developed to determine TC-NER activity for clinical and research purposes, monitoring TC-NER is hampered by the low frequency of repair events occurring in transcribed DNA. ’Recovery of RNA Synthesis’ is widely used as indirect TC-NER assay based on the notion that lesion-blocked transcription only resumes after successful TC-NER. Here, we show that measuring novel synthesis of a protein after its compound-induced degradation prior to DNA damage induction is an equally effective but more versatile manner to indirectly monitor DNA repair activity in transcribed genes. This ‘Recovery of Protein Synthesis’ (RPS) assay can be adapted to various degradable proteins and readouts, including imaging and immunoblotting. Moreover, RPS allows real-time monitoring of TC-NER activity in various living cells types and even in differentiated tissues of living organisms. To illustrate its utility, we show that DNA repair in transcribed genes declines in aging muscle tissue of *C. elegans*. Therefore, the RPS assay constitutes an important novel clinical and research tool to investigate transcription-coupled DNA repair.

## Graphical Abstract

**Graphical Abstract:**
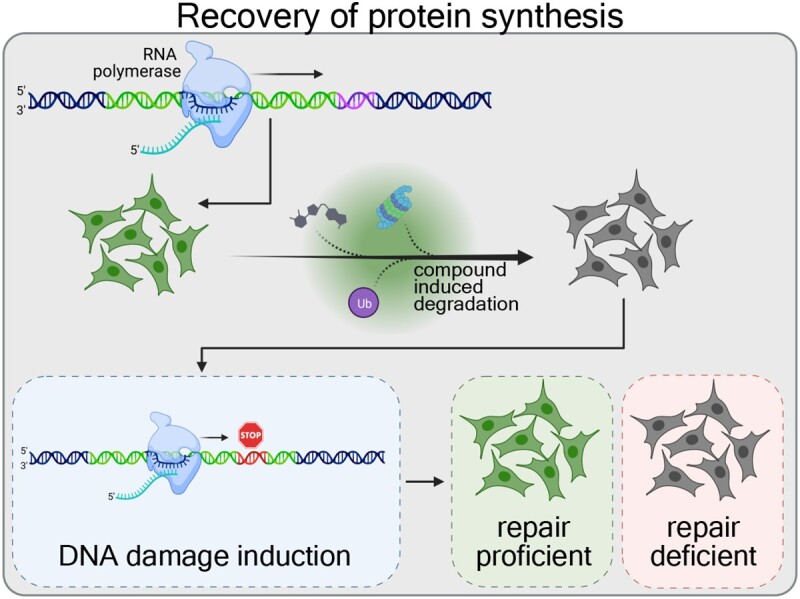

## INTRODUCTION

DNA damage induced by various environmental and metabolism-derived agents continuously threatens the integrity and functionality of DNA. DNA damage interferes with essential DNA-transacting processes like transcription and replication and contributes to aging and causes mutagenesis, leading to cancer (1). Various dedicated DNA repair pathways maintain genomic integrity by removing DNA damage depending on the type of lesion, its genomic location and the cell cycle stage. Nucleotide excision repair (NER) is an important DNA repair mechanism that protects organisms against cancer and aging by removing different DNA helix-distorting lesions, such as those induced by UV light, cancer therapeutics like cisplatin, metabolism-derived aldehydes and various genotoxic environmental chemicals including polycyclic aromatic hydrocarbon and aromatic amines found in smoke and cooked food (2,3).

NER is initiated by lesion detection via global genome NER (GG-NER) anywhere in the entire genome, or via transcription-coupled NER (TC-NER) exclusively in the transcribed strands of active genes. Lesion detection via GG-NER is mediated by the XPC protein, which continuously probes DNA and recognizes lesions by interacting with the DNA strand opposite of the lesion and inserting a β-hairpin domain into the distorted DNA duplex (4–6). XPC exists in complex with CETN2 and RAD23B and is aided by the CRL4^DDB2^ ubiquitin ligase complex when lesions are difficult to detect and/or when chromatin needs to be re-organized (7,8). Lesion detection via TC-NER occurs when lesions block forward progression of elongating RNA polymerase II (Pol II), which triggers the stable association of the CSB protein with Pol II (9). CSB then recruits the CRL4^CSA^ ubiquitin ligase complex and additional TC-NER factors (10). Lesion detection via either XPC or CSB leads to sequential recruitment of core NER factors, including TFIIH and XPA, which unwind DNA and check for the presence of damage, and the endonucleases ERCC1-XPF and XPG that excise a 22–30 bp DNA stretch containing the lesion (11,12). The resulting gap is then filled in by DNA synthesis mediated by replication factors, DNA polymerases and ligases.

The biological significance of NER is illustrated by the severe cancer-prone, developmental and progeroid symptoms associated with hereditary mutations in NER genes (13). GG-NER deficiency causes xeroderma pigmentosum (XP), which manifests as photosensitive skin and strong cancer predisposition (14). Mutations in TC-NER factors cause either the mild UV-sensitive syndrome (UV^S^S) or the much more severe Cockayne syndrome (CS) (15,16). Mutations in genes involved in both GG-NER and TC-NER can cause severe XP, often also combined with CS features, or a photosensitive form of trichothiodystrophy (TTD) (17,18). UV^S^S is mainly characterized by telangiectasia and sun sensitivity of the skin, whereas CS is characterized by a pleiotropic range of severe symptoms including growth failure, progressive organ decline and neurodegeneration and segmental progeria. It is currently not understood why different mutations in TC-NER factors lead to this wide array of symptoms, but it is hypothesized that this may be related to differences in clearance of lesion-stalled Pol II (19). TTD is characterized by brittle hair and nails, ichthyosis and progressive mental and physical retardation and is thought to be mainly caused by problems with gene expression (20). Interestingly, research on progeroid features of TC-NER deficiency disorders, in humans and mouse models, has revealed that accumulating DNA damage interfering with transcription is one of the major underlying causes of aging (21,22). To study the etiology of aging, and how this affects organs differently, it is therefore important to be able to investigate DNA repair activity in transcribed genes in different types of cells *in vivo*.

Both the clinical diagnosis of NER disorders as well as the discovery of novel genes involved in NER depends heavily on the availability of reliable and straightforward assays that can discriminate GG-NER and TC-NER deficiency. GG-NER activity can be determined by measuring unscheduled DNA synthesis (UDS) that restores the single-stranded DNA gap resulting from DNA damage excision. Originally, this assay was used to link DNA repair defects to XP and was based on GG-NER-dependent incorporation of radioactively labeled thymine analogs (23). Nowadays, these have been replaced by 5-ethynyl-2′-deoxyuridine, which can be visualized by fluorescent labeling using a click chemistry reaction (24,25). TC-NER activity is more difficult to detect largely due to the fact that this repair pathway only removes a minority of lesions, i.e. only those that block transcription. Originally, TC-NER was demonstrated using a Southern blot-based assay that showed preferential repair in the transcribed strands of active genes (26), which was subsequently used to show TC-NER deficiency in cells from CS patients (27). Since then, multiple assays have been developed to monitor TC-NER activity either directly, such as the gene-specific qPCR (28), comet-FISH (29), the amplified UDS in GG-NER deficient cells (30) and strand-specific ChIP-Seq (31) assays, or indirectly, such as the host cell reactivation (32) and the Recovery of RNA synthesis (RRS) assays. Because of its ease of use, the RRS assay is commonly used in NER research and in the clinic. It is based on the notion that DNA damage inhibits transcription and that this can only resume if TC-NER is active and removes the damage (33). Transcription recovery can be measured by labeling RNA with radioactive or bromo-uridine or, as is nowadays more common, 5-ethynyluridine that can be fluorescently labeled using click chemistry (24,25). A drawback of RRS and most other techniques is that these assays only work in cells in culture and cannot easily be used to determine repair activity in real-time or *in vivo*.

Here, we show the utility of a novel versatile assay to indirectly monitor DNA repair in transcribed genes based on the idea that not only transcription but also translation will only resume after DNA damage induction if DNA repair is functional. We show that this ‘recovery of protein synthesis’ (RPS) assay is robust and reliable and can be performed by monitoring the novel synthesis of different proteins, using both fluorescence imaging as well as immunoblotting as readout. Moreover, the RPS assay can be used to assay DNA repair activity in real-time both in living cells in culture as well as *in vivo* in differentiated cell types of a living organism.

## MATERIALS AND METHODS

### Cell culture and treatment conditions

Cell lines used are listed in Supplementary Table S1. U2OS (ATCC) and Hep3B cells stably expressing EGFP-AR (34,35) were cultured in DMEM (Invitrogen), supplemented with 10% fetal bovine serum (FBS; Bodinco BV), and 1% penicillin/streptomycin (Sigma). C5RO-SV40 (36), CS3BE-SV40 (GM16094; Coriell Institute) and CS1AN-SV40 (37) fibroblasts were cultured in HAM’s F10 (Invitrogen) supplemented with 15% FBS and 1% penicillin/streptomycin. Transient siRNA-mediated knockdown was achieved using transfection with Lipofectamine RNAiMAX (Invitrogen) according to the manufacturer's instruction. The siRNA oligonucleotides (Horizon Discovery; listed in Supplementary Table S2) used were: CTRL 5′-UGGUUUACAUGUUGUGUGA-3′, CSB 5′-GCAUGUGUCUUACGAGAUA-3′, XPF 5′-AAGACGAGCUCACGAGUAU-3′ and XPC 5′-GCAAAUGGCUUCUAUCGAA-3′. Knockdown was confirmed by immunoblot (Supplementary Figure S1A). Cells were incubated with 50 nM dTAG-13 (Tocris) to degrade EGFP-FKBP^F36V^, 100 nM ARV-110 (Selleckchem) to degrade EGFP-AR and 250 nM PROTAC FAK degrader 1 (MedChemExpress) to degrade PTK2/FAK, as indicated. Nuclear localization of EGFP-AR was induced by incubating cells with 0.028 μg/ml R1881. XPB depletion was achieved by 4 h spironolactone (Sigma) incubation with the indicated doses. For induction of DNA damage by UV, cells were exposed to 6 J/m^2^ UV-C light (254 nm; TUV lamp, Phillips). For cisplatin induction of DNA damage, cells were exposed for 2 h to 100 μM cisplatin (Sigma). All reagents used in this study, with concentrations and identifiers, are listed in Supplementary Table S3.

### Plasmid and cell line generation

To generate U2OS cells stably expressing EGFP-FKBP^F36V^, a gene fragment containing *EGFP*-*FKBP^F36V^* with nuclear localization signal behind a PGK promoter and *BLAST* behind an EF1a promoter, flanked by *AAVS1* homology sequences for homology directed repair, was generated by Integrated DNA technologies. EGFP sequence was derived from pEGFP-N1 (Clontech), FKBP^F36V^ sequence was derived from pLEX_305-C-dTAG (Addgene #91798) (38) and *BLAST* sequence was derived from pLenti-PGK-GFP-Blast (Addgene #19069) (39). The gene fragment was cloned into pUC57 (Genscript) and transfected together with Cas9 (IDT) and an sgRNA targeting AAVS1 (GGGGCCACTAGGGACAGGAT; IDT) into U2OS cells. Transfection was performed with Lipofectamin CRISPRMAX (Thermofisher) according to the manufacturer's instructions. After selection with blasticidin, a clonal cell line stably expressing EGFP-FKBP^F36V^ was isolated. The EGFP-FKBP^F36V^ sequence and plasmid are available from Addgene (Addgene plasmid PGK-EGFP-FKBPF36V #199765).

### Live-cell confocal laser-scanning microscopy

For live cell imaging, cells were grown on 24-mm coverslips and imaged using a Leica SP5 confocal microscope equipped with an environmental chamber set to 37°C and 5% CO2. Confocal images were recorded every hour after UV irradiation, as indicated. Data collection and analysis was performed using LAS X software (Leica).

### Fluorescence imaging

For imaging of fluorescence levels, cells were grown on 24-mm coverslips and fixed using 3.6% formaldehyde (Sigma) diluted in PBS. 4′,6-diamidino-2-phenylindole (DAPI; Sigma) staining was performed in PBS for 15 min at room temperature. Subsequently, coverslips were washed twice with PBS supplemented with 0.1% Triton-X-100 and once with PBS and mounted with aqua-poly/mount (Polysciences Inc). Cells were imaged using a Zeiss LSM700 microscope equipped with a Plan-Apochromat 40x/1.3 Oli DIC M27 immersion lens (Carl Zeiss Microimaging Inc.). EGFP-FKBP^F36V^ or EGFP-Androgen receptor expression in the nucleus was quantified in Fiji ImageJ.

### Immunoblot and antibodies

For immunoblotting, cells were lysed in sample buffer (0.125 M Tris–HCl pH 6.8, 2% SDS, 0.005% bromophenol blue, 21% glycerol, 4% β-mercaptoethanol) and boiled for 5 min at 98°C. Lysates were separated by sodium dodecyl sulphate-polyacrylamide gel electrophoresis (SDS-PAGE) using NuPAGE™ 4–12% Bis–Tris gels (Invitrogen) and transferred to polyvinylidene difluoride (PVDF) membrane (0.45 μm) (Millipore). Membranes were blocked in 5% BSA (Sigma) for 30 min at room temperature and incubated for 2 h at room temperature or overnight at 4°C with primary antibodies against GFP (Roche), androgen receptor (Invitrogen), PTK2 (Invitrogen), XPB (Abcam), CSB (Bethyl), XPF (Santa Cruz), XPC (Bethyl), Tubulin (Sigma Aldrich) or Ku70 (Santa Cruz). Membranes were incubated with secondary antibodies coupled to IRDyes (Sigma) and scanned using an Odyssey CLx infrared scanner (LiCor). Antibodies, identifiers and concentrations are listed in Supplementary Table S4.

### *C. elegans* strains and experimental handling

*Caenorhabditis elegans* (*C. elegans*) were cultured according to standard methods (40). Strains and alleles used were CA1202 (*ieSi57[P(eft-3)::TIR1::mRuby] II; ieSi58 [P(eft-3)::AID::GFP] IV*) (41); HAL526 (*ieSi57 [P(eft-3)::TIR1::mRuby] II; ieSi58 [P(eft-3)::AID::GFP] IV; csb-1(ok2335)*); HAL534 (*ieSi57 [P(eft-3)::TIR1::mRuby] II; ieSi58 [P(eft-3)::AID::GFP] IV; xpc-1(tm3886)*); HAL535 (*ieSi57 [P(eft-3)::TIR1::mRuby] II; ieSi58 [P(eft-3)::AID::GFP] IV; csb-1(ok2335); xpc-1(tm3886)*), as also listed in Table S1. HAL526, HAL534 and HAL535 strains were generated by crossing CA1202 with *xpc-1(tm3886)* and/or *csb-1(ok2335)* mutants (42) and genotyped by PCR and sequencing. For the recovery of protein synthesis assay, animals of similar age were depleted of AID::GFP expression by culturing for 2 h on NGM plates containing 100 μM auxin (3-indoleacetic acid; Sigma). Directly after depletion, animals were mock-treated or irradiated with 120 J/m^2^ UV-B (Philips TL-12 tubes, 40W) and allowed to recover for 48 h on NMG plates containing OP50 *E. coli* food. AID::GFP levels were measured by imaging fluorescence in body wall muscle cells in the head of living animals on a Leica TCS SP8 microscope (LAS AF software, Leica). AID::GFP expression levels were quantified in Fiji ImageJ.

### Statistical analysis and software

Software used is listed in Supplementary Table S5. Statistical analyses were performed using Graph Pad Prism version 9 for Windows (GraphPad Software, La Jolla California USA). In each graph, mean values and S.E.M. error bars are shown and the number of experiments and cells or *C. elegans* tested is indicated in the legend. All underlying data is reported in Supplementary Table S6. For the live-cell imaging assays an unpaired t-test was used. For all other experiments, we applied a one-way ANOVA with correction for multiple comparison.

## RESULTS AND DISCUSSION

### Recovery of protein synthesis after DNA damage depends on transcription-coupled NER

**Figure 1.**
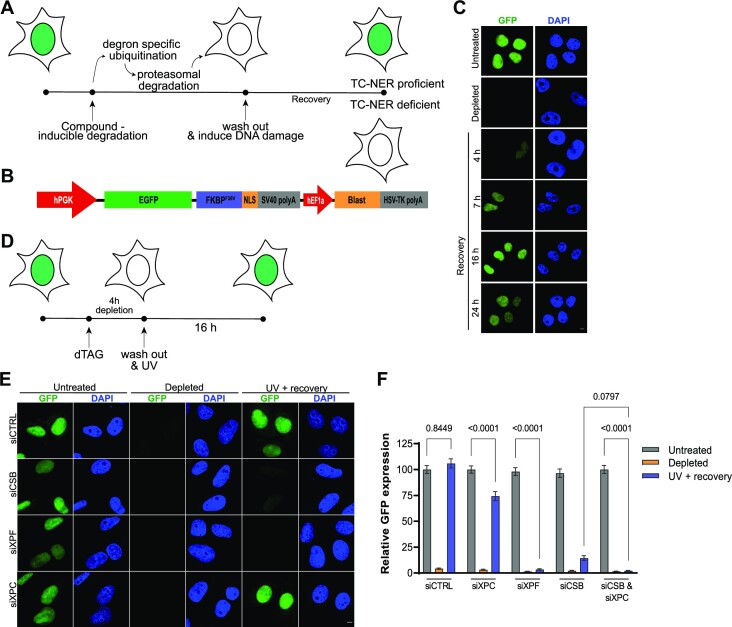
Recovery of protein synthesis after DNA damage depends on transcription-coupled NER**. (A)** Schematic depiction of the rationale of the Recovery of Protein Synthesis (RPS) assay. First a protein (indicated by green shading in the nucleus) is degraded in cells, after which cells are exposed to a DNA damaging agent and the novel synthesis of the protein is monitored in time. The protein will only be produced in TC-NER proficient cells after DNA damage induction. **(B)** Scheme of the transgene knocked-in at the AAVS1 locus to drive EGFP-FKBP^F36V^ from the PGK promoter and blasticidin (for selection) from the hEF1a promoter. **(C)** Representative images of fixed U2OS cells stably expressing EGFP-FKBP^F36V^, before treatment (untreated) and directly (depleted) or several hours (as indicated) after treatment with dTAG13. **(D)** Timing scheme of the RPS assay using UV-C irradiation. **(E)** Representative images of fixed EGFP-FKBP^F36V^-expressing U2OS cells transfected with control siRNA (siCtrl) or siRNA against CSB (siCSB), XPF (siXPF) or XPC (siXPC) that were either untreated, incubated with dTAG13 for 4 h (‘depleted’) or incubated with dTAG13 and irradiated with 6 J/m^2^ UV-C and left to recover for 16 h (‘UV + recovery’). Scale bars 5 μm **(F)** Quantification of GFP expression levels from siRNA-treated cells imaged and treated as explained in **(E)**. Bars depict the mean with S.E.M. of individual cells measured in three independent experiments. The number of cells measured in respective order shown in the graph is 292, 274, 217, 257, 275, 181, 398, 250, 216, 326, 238, 260, 292, 291 and 206. Statistical differences were determined by one-way ANOVA with correction for multiple comparison. Numbers in the graph indicate p-values. Source data for the graph can be found in Supplementary Table S6.

As the RRS assay is based on measuring the ability of cells to recover transcription after DNA damage induction, we reasoned that the ability of cells to re-synthesize a degraded protein could be used as indirect TC-NER readout as well (Figure 1A). After protein degradation, ongoing transcription will produce new proteins, which will still happen if cells incur transcription-blocking DNA damage that is repaired. However, in the absence of TC-NER, transcription blockage will persist and less or no new protein will be produced. We first tested this idea using GFP, as imaging the re-synthesis of a fluorescent reporter will allow the monitoring of TC-NER activity in real-time in living cells. We therefore generated a DNA construct expressing EGFP fused to the FKBP^F36V^ degradation tag (dTAG) (38) and to a nuclear localization signal, which can be efficiently knocked-in at the AAVS1 locus using CRISPR-Cas9 in any cell type (43) and selected for by blasticidin (Figure 1B). Proteins fused to the mutant FKBP^F36V^ dTAG are rapidly degraded by the proteasome via polyubiquitination by the CRL4^Cereblon^ E3 ubiquitin ligase complex, after addition of a heterobifunctional dTAG ligand such as dTAG-13 to cells (38). Rapid protein depletion using this system before DNA damage induction will therefore enable us to determine if, after DNA damage induction, novel protein is synthesized and therefore DNA repair has taken place. After establishing human osteosarcoma U2OS cells stably expressing EGFP-FKBP^F36V^ in the nucleus, we found by fluorescence imaging of fixed cells that addition of dTAG-13 led to rapid loss of the nuclear fluorescent signal, which re-appeared upon washing away dTAG-13 (Figure 1C).

Subsequently, we tested if EGFP protein synthesis is dependent on TC-NER after DNA damage induction. We treated the cells with control siRNA or siRNA against the GG- and TC-NER factor XPF, the GG-NER factor XPC and/or the TC-NER factor CSB, after which we depleted EGFP. We first tested re-appearance of EGFP-FKBP^F36V^ expression in the absence of UV irradiation and observed that this was similarly robust in all siRNA-treated cells (Supplementary Figure S1B). Hereafter, we UV irradiated the cells and determined EGFP-FKBP^F36V^ protein synthesis recovery (Figure 1D). Untreated cells, which were not exposed to dTAG-13 and UV-irradiation, were used for comparison. In control siRNA-treated cells, the EGFP fluorescence levels returned to similar levels as in untreated cells within 16 h (Figure 1E and F). In sharp contrast, EGFP protein synthesis was completely abolished in XPF-depleted cells and almost completely abolished in CSB-depleted cells. Depletion of XPC led to a minor reduction in EGFP levels and further reduced EGFP levels in CSB-depleted cells to the same level as in XPF-depleted cells. These results demonstrate that protein resynthesis after its depletion can be used as readout of TC-NER capacity after DNA damage induction.

Our results suggest that in UV-irradiated cells GG-NER via XPC is responsible for repairing a minor fraction of lesions in transcribed genes, indicating that this method not only monitors TC-NER activity but any type of DNA repair activity that removes transcription-blocking DNA lesions. UV-C irradiation mainly induces (6–4) photoproducts (6–4PPs) and cyclobutane pyrimidine dimers (CPDs). These lesions are rapidly removed with equal efficiency by TC-NER (44), but CPDs are much less efficiently recognized and slower repaired by GG-NER than by TC-NER (5,45). The small fraction of XPC-dependent repair therefore likely reflects GG-NER of 6–4PPs rather than CPDs. Strikingly, using a moderate dose of 6 J/m^2^ UV-C irradiation, we observed a persisting complete depletion of the EGFP-FKBP^F36V^ protein in NER deficient cells, indicating that transcription of the encoding transgene was fully inhibited. This UV dose is estimated to produce on average less than one lesion per 10 000 bp (44), suggesting that our EGFP-FKBP^F36V^ transgene of 1122 bp is not damaged in each cell. It is therefore not likely that Pol II is physically blocked by DNA damage in the EGFP-FKBP^F36V^ transgene in every cell measured. Indeed, transcription shutdown in response to DNA damage does not only occur *in cis* due to direct physical blockage of elongating Pol II in a particular gene, but also takes place *in trans*, i.e. through genome-wide inhibition of Pol II-mediated transcription initiation and elongation (46–50). Therefore, the lack of EGFP protein synthesis after this moderate UV irradiation of cells with depletion of XPF and CSB most likely reflects the genome-wide shutdown of transcription due to DNA damage induction in a subset of genes. Because of this, it is possible to use the translation of even a relatively short gene like the EGFP-FKBP^F36V^ transgene to monitor DNA damage-induced transcriptional responses and the activity of TC-NER or other repair pathways that remove transcription-blocking lesions. In analogy to the RRS assay, we term our assay therefore ‘Recovery of Protein Synthesis’ (RPS).

### Recovery of protein synthesis monitors TC-NER capacity in real-time in living cells

**Figure 2.**
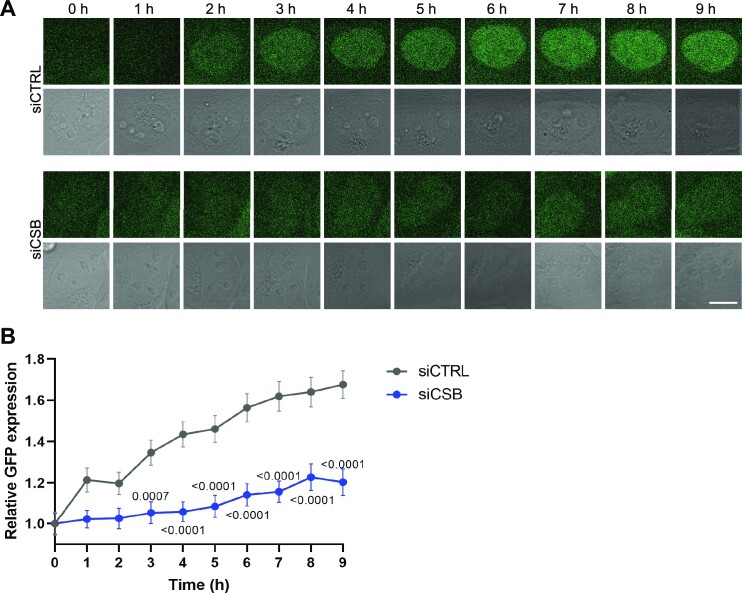
Recovery of protein synthesis monitors TC-NER activity in living cells. **(A)** Representative live cell imaging pictures of EGFP-FKBP^F36V^-expressing U2OS cells transfected with control (siCtrl) or CSB (siCSB) siRNA. Cells were incubated with dTAG13 for 8 h, irradiated with 6 J/m^2^ UV-C and imaged every hour as indicated. Scale bars, 10 μm **(B)** Quantification of GFP signal of EGFP-FKBP^F36V^-expressing U2OS cells treated and imaged as explained in **(A)**. Depicted is the mean and S.E.M. of individual cells measured in two independent experiments. The number of measured cells in respective order shown in the graph is 108, 125, 133, 138, 142, 136, 146, 129, 118 and 119 for siCTRL, and 98, 95, 99, 98, 100, 98, 96, 110, 100 and 89 for siCSB. Statistical differences were determined with unpaired t-test. Numbers in the graph indicate p-values. Source data for the graph can be found in Supplementary Table S6.

After observing that the RPS assay can be performed by imaging cells that were fixed at defined time points, we tested if RPS can similarly be monitored in real-time in living cells. We therefore imaged cells treated with siRNA and depleted of EGFP-FKBP^F36V^ for 9 h by live cell confocal microscopy. EGFP fluorescence signal recovered similarly in cells that were not UV irradiated and treated with either control or CSB siRNA (Supplementary Figure S2). In UV irradiated cells treated with control siRNA, the fluorescent EGFP signal recovered also already visibly within 1–2 h and continued to increase for 9 h without reaching plateau levels yet. In contrast, in CSB-depleted cells, hardly any fluorescence signal was observed after 1–2 h and only a low recovery of fluorescence signal was visible at later time points (Figure 2A and B; Supplementary movies S1 and S2). These results indicate that measuring RPS in living cells reflects real-time recovery of gene expression and thus TC-NER activity. In future studies, it might therefore be possible to use the RPS assay to determine and compare TC-NER activity between individual cells, which could for instance be combined with single cell sequencing or proteomics techniques to determine if any observed differences have a molecular (epi)genetic and/or proteomic basis (51).

### Recovery of protein synthesis can be performed with chemicals that affect TC-NER

**Figure 3.**
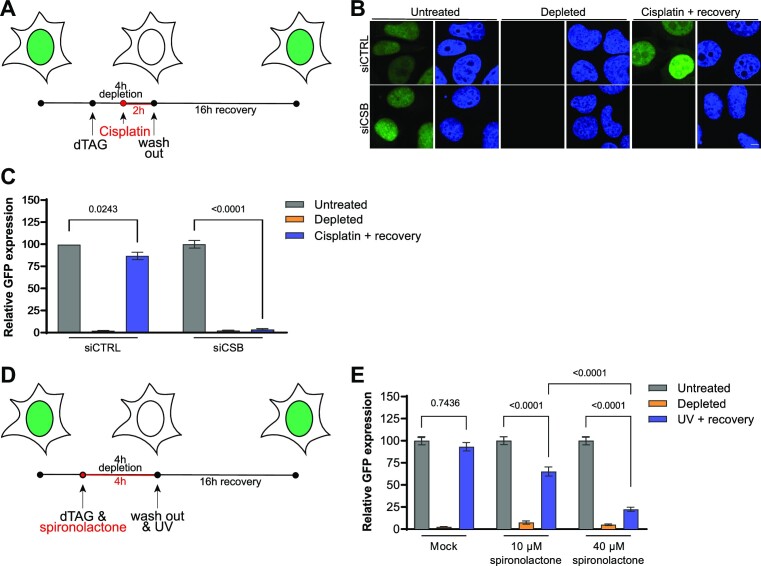
Recovery of protein synthesis after cisplatin or chemical NER inhibition. **(A)** Timing scheme of the Recovery of Protein Synthesis assay using cisplatin. **(B)** Representative images of fixed EGFP-FKBP^F36V^-expressing U2OS cells transfected with control (siCtrl) or CSB (siCSB) siRNA that were either untreated, incubated with dTAG13 for 4 h (‘depleted’) or with dTAG13 for 4 h and 100 μM cisplatin for 2 h and left to recover for 16 h (‘Cisplatin + recovery’). Scale bars, 5 μm **(C)** Quantification of GFP expression levels from siRNA-treated cells imaged and treated as explained in **(B)**. Mean and S.E.M. of three independent experiments. **(D)** Timing scheme of the Recovery of Protein Synthesis assay using spironolactone. **(E)** Quantification of GFP expression levels from mock or spironolactone-treated EGFP-FKBP^F36V^-expressing U2OS cells that were either untreated, incubated with dTAG13 for 4 h (‘depleted’) or with dTAG13 for 4 h and then irradiated with 6 J/m^2^ UV-C and left to recover for 16 h (‘UV + recovery’). Bars depict the mean with S.E.M. of individual cells measured in three independent experiments. The number of cells measured in respective order shown in the graph is **(C)** 288, 310, 250, 330, 251 and 199. **(E)** 362, 344, 263, 391, 320, 191, 334, 257 and 192. Statistical differences were determined by one-way ANOVA with correction for multiple comparison. Numbers in the graphs indicate p-values. Source data for the graphs can be found in Supplementary Table S6.

To determine if RPS accurately reflects TC-NER activity in response to DNA damaging agents other than UV irradiation, we exposed siRNA-treated and EGFP-depleted cells to cisplatin for 2 h (Figure 3A). Cisplatin is a commonly used chemotherapeutic drug that mainly creates 1,2-d(GpG) and 1,2-d(ApG) intrastrand crosslinks that effectively inhibit transcription and are repaired via TC-NER (52–55). Similar as after UV irradiation, we observed that the EGFP fluorescence signal in control siRNA-treated cells recovered after cisplatin exposure (Figure 3B and C). In contrast, no fluorescence recovery was observed at all in CSB-depleted cells after cisplatin exposure. These results illustrate the versatile utility of the RPS assay to determine TC-NER and transcription restart activity in response to different types of genotoxic insults.

We subsequently tested if the RPS assay is compatible with pharmacological inhibition of NER (Figure 3D). We exposed EGFP-depleted cells for 4 h to spironolactone, which induces rapid and reversible degradation of TFIIH subunit XPB (Supplementary Figure S3A) and therefore interferes with NER and transcription (56). Spironolactone did not strongly delay RPS in cells that were not exposed to UV irradiation, despite the importance of XPB for transcription initiation (Supplementary Figure S3B). However, increasing concentrations of spironolactone led to a correspondingly stronger RPS defect in UV irradiated cells (Figure 3E and Supplementary Figure S3B, which depict two independent sets of experiments). This shows that the RPS assay can be effectively used to monitor the impact of chemical inhibitors on DNA repair of transcription-blocking lesions. Together, these results indicate that the RPS assay is likely very suited for screening purposes. RPS could be used to screen for genotoxic compounds that block transcription and require TC-NER to overcome the transcription blockage, like cisplatin. Moreover, this type of screening could be combined with high-content screening approaches using genomic siRNA, shRNA or sgRNA libraries to identify the factors involved in repair of these lesions. Additionally, RPS could be used in high-content screening approaches, using fixed or living cells, to screen for compounds that inhibit transcription-coupled DNA repair.

### RPS can be used as diagnostic tool for TC-NER activity with any degradable protein

**Figure 4.**
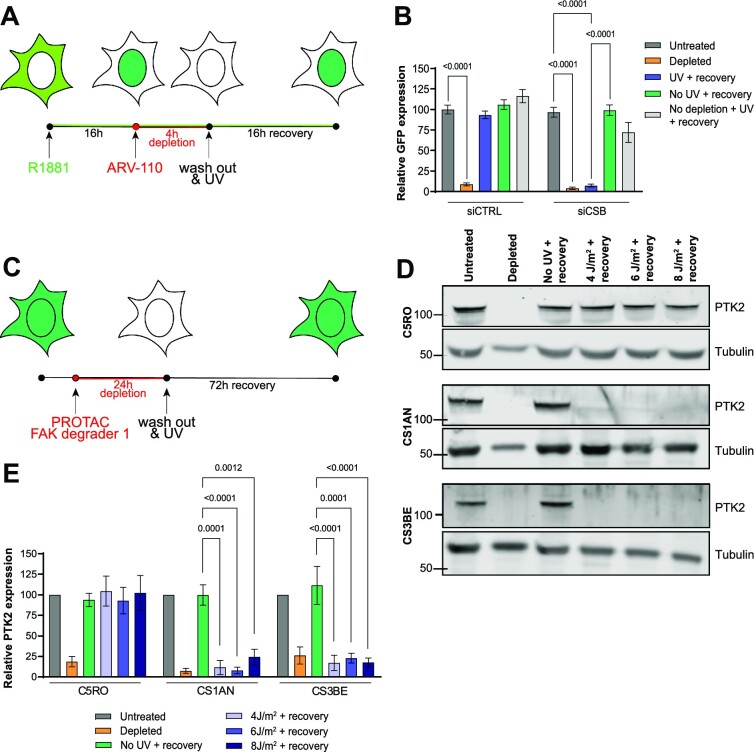
Novel synthesis of PROTAC-degraded proteins to monitor TC-NER. **(A)** Timing scheme of the Recovery of Protein Synthesis assay using ARV-110. **(B)** Quantification of GFP fluorescence levels in EGFP-AR-expressing Hep3B cells that were either untreated or incubated with 100 nM ARV-110 for 4 h (‘depleted’), incubated with 100 nM ARV-110 for 4 h and then irradiated with 6 J/m^2^ UV-C and left to recover for 16 h (‘UV + recovery’), incubated with 100 nM M ARV-110 for 4 h and left to recover for 16 h (‘no UV + recovery’) or irradiated with 6 J/m^2^ UV-C and left to recover for 16 h (‘No depletion + UV + recovery’). Bars depict the mean with S.E.M. of individual cells measured in three independent experiments. The number of cells measured in respective order shown in the graph is **(B)** 181, 157, 184, 159, 115, 163, 161, 211, 179 and 55. **(C)** Timing scheme of the Recovery of Protein Synthesis assay using PROTAC FAK degrader 1. **(D)** Immunoblot analysis of cell lysate from SV40-immortalized C5RO, CS1AN and CS3BE cells that were either untreated or incubated with 250 nM PROTAC FAK degrader 1 for 24 h (‘depleted’), or incubated with 250 nM PROTAC FAK degrader 1 for 24 h and irradiated with the indicated UV dose and left to recover for 72 h (‘indicated UV dose + recovery’). Immunoblots are stained with antibodies against PTK2 and Tubulin (as loading control). **(E)** Quantification of protein levels based on immunoblots as shown in (D). Mean and S.E.M. of four (C5RO) or three (CS1AN and CS3BE) independent experiments. Statistical differences were determined by one-way ANOVA with correction for multiple comparison. Numbers in the graphs indicate p-values. Source data for the graphs can be found in Supplementary Table S6.

After establishing the RPS assay by monitoring novel synthesis of dTAG-mediated degraded EGFP in U2OS cells, we tested if instead also other proteins and/or other cell types, including patient-derived fibroblasts, can be used for RPS. We therefore first attempted RPS by monitoring expression of the androgen receptor (AR) after its depletion with Bavdegalutamide (ARV-110) (Figure 4A). ARV-110 is a PROTAC degrader that induces AR ubiquitination and subsequent degradation (57). To easily quantify AR levels, we used human hepatoma Hep3B cells stably overexpressing AR fused to EGFP and incubated with the synthetic AR ligand R1881 that induces nuclear localization of AR (34,35). Imaging showed that EGFP-AR protein levels were already slightly reduced 16 h after UV irradiation in CSB-depleted cells without the addition of ARV-110 to cells. Even more so, we observed that after ARV-110 addition, the EGFP-AR protein levels only clearly recovered in cells treated with control siRNA, but not in cells treated with siCSB (Figure 4B).

We subsequently tested if we could similarly use this PROTAC-mediated degradation of endogenously-expressed non-tagged proteins to perform RPS in human fibroblasts of CS patients, using immunoblotting instead of fluorescence imaging. As the AR protein is not clearly expressed in fibroblasts (Supplementary Figure S3C), we instead monitored RPS of the PTK2/FAK protein (Figure 4C), which can be efficiently degraded by exposure to the PROTAC FAK degrader 1 (58). We depleted PTK2 in TC-NER proficient control C5RO fibroblasts and in fibroblasts derived from CSB-deficient (CS1AN) and CSA-deficient (CS3BE) CS patients (59). Immunoblotting of cell lysates from these fibroblasts after UV-irradiation showed that PTK2 protein synthesis only clearly recovered in the TC-NER proficient control cells (Figure 4D and E). These results indicate that monitoring RPS of any protein that can be inducibly depleted or degraded, in any cell type of choice, can be used to monitor TC-NER activity and/or transcription recovery after DNA damage induction. Moreover, the successful application of RPS in patient fibroblasts indicates that this assay could be useful for clinical diagnosis of CS, as versatile alternative to RRS. As we noticed that resynthesis of PTK2 requires substantially longer time than resynthesis of ectopically expressed AR, it will be useful to test and compare additional PROTACs and their protein targets to determine which are most efficient and cost-effective for potential future (clinical) applications of the RPS assay.

### RPS monitors DNA repair of transcription-blocking lesions in young and old tissues of living organisms

**Figure 5.**
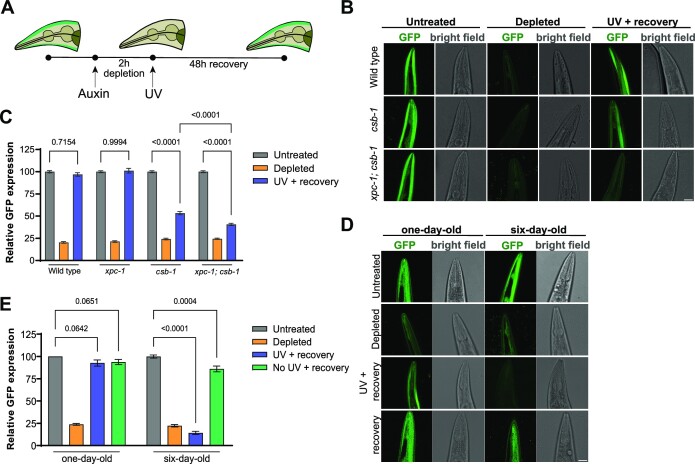
Recovery of protein synthesis assay in muscle cells of *C. elegans*.**(A)** Timing scheme of the Recovery of Protein Synthesis assay using UV-irradiated *C. elegans*. Depicted is a schematic drawing of the head of *C. elegans* with body wall muscle cells expressing green colored GFP. **(B)** Representative confocal images of living wild type, *csb-1* and *xpc-1; csb-1* animals expressing AID::GFP (and TIR1::m-Ruby, not depicted) under control of the *eft-3* promoter in body wall muscles, shown here in the head of *C. elegans*. Animals were either untreated, cultured on 100 μM auxin for 2 h (‘depleted’) or cultured on 100 μM auxin for 2 h and then irradiated with 120 J/m^2^ UV-B and left to recover for 48 h (‘UV + recovery’). Scale bar, 25 μm. **(C)** Quantification of GFP fluorescence levels in muscle cells of wild type, *xpc-1, csb-1* and *xpc-1; csb-1* animals expressing AID::GFP and treated and imaged as explained in (B). Mean and S.E.M. of three independent experiments. **(D)** Representative confocal images of living one-day old and six-day old wild type animals expressing AID::GFP in body wall muscles, shown here in the head of *C. elegans*. Animals were either untreated, cultured on 100 μM auxin for 2 h (‘depleted’), cultured on 100 μM auxin for 2 h and then irradiated with 120 J/m^2^ UV-B and left to recover for 48 h (UV + recovery) or cultured on 100 μM auxin for 2 h and left to recover for 48 h (‘no UV + recovery’). Scale bar, 25 μm. **(E)** Quantification of GFP fluorescence levels in muscle cells of one-day-old and six-day-old wild type animals expressing AID::GFP and treated and imaged as explained in (D). Bars depict the mean with S.E.M. of individual cells in *C. elegans* measured in three independent experiments. In each independent experiment at least three animals were tested. The number of cells measured in respective order shown in the graph is (C) 229, 138, 207, 178, 83, 167, 275, 151, 209, 263, 183 and 310 (E) 85, 75, 84, 70, 86, 68, 88 and 66. Statistical differences were determined by one-way ANOVA with correction for multiple comparison. Numbers in the graphs indicate p-values. Source data for the graphs can be found in Supplementary Table S6.

To test if the RPS assay can be applied *in vivo*, in differentiated cells of a living organism, we tested if novel protein synthesis in UV irradiated muscle cells of *C. elegans* depends on TC-NER activity (Figure 5A). NER is well-conserved in *C. elegans* and particularly TC-NER is active in differentiated somatic tissues (60,61). XPC-1 and CSB-1 are *C. elegans* orthologs of human XPC and CSB, respectively. We generated transgenic wild type and XPC-1- and CSB-1-deficient animals expressing GFP fused to an auxin-inducible degradation tag (AID::GFP) and *Arabidopsis* TIR1 (fused to mRuby) (41) under control of the *eft-3* promotor driving ubiquitous expression including in muscle cells. TIR1 forms an E3 ubiquitin ligase complex that can be activated by culturing animals on the auxin plant hormone indole-3-acetic acid, which leads to ubiquitylation of the AID tag and subsequent proteasomal degradation of AID::GFP in body wall muscle cells (Figure 5B). 48 h after UV irradiation of *C. elegans* cultured on auxin for 2 h (Figure 5A), we imaged living animals by confocal microscopy and observed that in wild type and XPC-deficient animals the AID::GFP fluorescence had returned to the same level as that of animals not treated with auxin (Figure 5B and C). Contrarily, AID::GFP fluorescence levels in UV-irradiated CSB-1-deficient animals remained strongly reduced. Previous survival experiments had suggested that XPC-1 can partially compensate for the lack of repair in active genes in somatic cells of TC-NER-deficient *C. elegans* (42,62). In line with this, we observed that additional loss of XPC-1 in CSB-1-deficient animals further reduced AID::GFP fluorescence levels after UV. These results confirm that XPC-1 acts partially redundant to CSB-1, as was also observed in human cells (Figure 1E and F).

As the RPS assay accurately reflects the repair of transcription-blocking DNA damage in active genes in muscle cells, we wondered whether it can be used to reveal changes in DNA repair activity when animals grow older, as DNA repair is proposed to decline with age (63). We therefore compared AID::GFP protein levels after UV irradiation in one-day-old wild type adult animals to those in six-day old animals. Whereas UV-irradiated one-day-old animals and unirradiated one-day-old and six-day-old animals all fully recovered GFP fluorescence levels after culturing on auxin, strikingly, UV-irradiated six-day-old animals showed no fluorescence recovery at all (Figure 5D and E). These results suggest that aging *C. elegans* lose their ability to repair DNA damage in actively transcribed genes, at least in muscle cells. These results are in line with previously observed reduced repair in six-day-old animals using gene-specific qPCR assays (64). Together, our results show that the RPS assay can be applied in living, multicellular organisms to compare the capacity to remove transcription-blocking lesions, such as by TC-NER, between tissues, different genetic backgrounds and/or between developmental stages or during aging. It will be interesting to investigate if a similar reduction of transcription-associated DNA repair capacity can be determined in aging tissues of e.g. mouse models of aging, as accumulating DNA damage leading to transcription stress is considered one of the driving forces of the aging process (22,65). For such a purpose, variations to the RPS method may be used or may be further developed. A similar principle as applied here, i.e. measuring the ability of protein expression, was previously used in *C. elegans* to show that UV-induced developmental arrest in XPA deficient animals is due to transcription blockage (66). Instead of using degradable GFP, the ability of UV-irradiated animals to express heat-shock promoter-driven GFP was tested. Although it may not be straightforward to use heat-shock inducible promoters in mammalian cells or model systems, besides degradable proteins alternatively other inducible transcriptional reporter systems could be used to measure the ability of protein synthesis as read-out for TC-NER capacity.

### Prospects and limitations of the RPS assay

Our results indicate that the RPS assay is an equally effective method as the widely used RRS to monitor the capacity of cells to remove transcription-blocking DNA damage from genes and/or to restart transcription after DNA damage induction. Both the protein synthesis recovery in control cells as well as the lack of recovery in TC-NER deficient cells are equally robust and reproducible as transcription recovery measured by RRS, such as e.g. previously determined by RRS in our laboratory in control and TC-NER deficient cells using similar UV (6 J/m^2^) or cisplatin (100 μM) doses (52,67). The RPS assay, however, possesses a greater degree of versatility than RRS, because various read-outs such as imaging and western blotting can be used, and especially because protein recovery can be imaged in real-time in living cells and *in vivo* as well. Moreover, because the fluorescence signal of degron-tagged GFP is monitored directly in fixed or living cells, without the need of a click-chemistry reaction to couple fluorescent azide to 5-ethynyluridine as in RRS (24,25), the RPS assay is essentially easier to perform and possibly better compatible with immunofluorescence of other proteins. Another advantage and versatile aspect of the RPS assay is that it will likely work with any protein that can be degraded, either using a degron tag or a PROTAC degrader. Therefore, different adaptations of the assay will be best suitable for different purposes. For instance, recovery of degron-tagged GFP in combination with siRNA-mediated knockdown of proteins is suited to rapidly test if a protein of interest may be involved in repair of transcription-blocking DNA damage. This version of the assay will therefore also be suited for genetic screening purposes. Alternatively, it may be possible to bleach the GFP signal instead of degrading the fluorescent protein, and image the recovery of fluorescence to monitor DNA repair activity, which may be easier for use in some *in vivo* applications. Additionally, the compound-induced degradation of a relatively long gene may be more suited for use in an RPS assay that needs to be sensitive to low doses of DNA damage, as a longer gene will incur more DNA damage. For diagnostic purposes, however, the use of PROTAC degraders and immunoblotting will likely be an easy and suitable approach, as this can be applied by laboratories that do not have adequate imaging facilities as well.

Several proteins and histone modifications have been implicated in transcription restart following DNA damage induction and DNA repair, rather than DNA repair itself (68–72). It is important to emphasize that the RPS assay, although versatile in its applicability with different cell types both *in vitro* and *in vivo*, cannot be used to distinguish between TC-NER and restart defects. To make such a distinction, results should be compared to those obtained in assays directly measuring TC-NER such as the amplified UDS in GG-NER deficient cells (30) or strand-specific ChIP-Seq (31) assays. Also, it is unclear to what extent translation itself is affected by the DNA damage doses that we used. We did not clearly observe new protein synthesis after depletion of GFP for 4 h in completely NER-deficient cells, suggesting that also no translation took place from mRNA transcripts that already existed before DNA damage induction. This is striking as GFP mRNA, and thus possibly also the mRNA of GFP tagged with a degron, has a half time of more than 4 h (73). Therefore, this suggests that besides transcription also protein synthesis is inhibited, as also previously reported to occur after UV irradiation, even though this was observed after relatively high UV doses (74–78). Additionally, translation may be inhibited as a result of reduced expression of genes encoding protein synthesis factors, as observed in *C. elegans* after UV irradiation (72). Thus, possibly, our assay could be useful to identify factors involved in regulating the translational response to DNA damage as well. As the RPS assay is easily adjustable for use with different cell types, degradable target proteins and/or experimental readouts, it is expected that the assay will be very useful for future screening purposes utilizing living cells or even tissue models such as organoids or various model organisms.

## Supplementary Material

gkad642_Supplemental_Files

## Data Availability

The EGFP-FKBP^F36V^ plasmid was deposited at Addgene (#199765). Other plasmids, cell lines and *C. elegans* strains generated in this study are available upon reasonable request.

## References

[1] Hoeijmakers J.H.J. DNA damage, aging, and cancer. N. Engl. J. Med. 2009; 361:1475–1485. 19812404 10.1056/NEJMra0804615

[2] Gillet L.C.J. , Schärer O.D. Molecular mechanisms of mammalian global genome nucleotide excision repair. Chem. Rev. 2006; 106:253–276. 16464005 10.1021/cr040483f

[3] Mulderrig L. , Garaycoechea J.I., Tuong Z.K., Millington C.L., Dingler F.A., Ferdinand J.R., Gaul L., Tadross J.A., Arends M.J., O’Rahilly S. et al. Aldehyde-driven transcriptional stress triggers an anorexic DNA damage response. Nature. 2021; 600:158–163. 34819667 10.1038/s41586-021-04133-7

[4] Paul D. , Mu H., Zhao H., Ouerfelli O., Jeffrey P.D., Broyde S., Min J.H. Structure and mechanism of pyrimidine-pyrimidone (6-4) photoproduct recognition by the Rad4/XPC nucleotide excision repair complex. Nucleic Acids Res. 2019; 47:6015–6028. 31106376 10.1093/nar/gkz359 PMC6614856

[5] Sugasawa K. , Okamoto T., Shimizu Y., Masutani C., Iwai S., Hanaoka F. A multistep damage recognition mechanism for global genomic nucleotide excision repair. Genes Dev. 2001; 15:507–521. 11238373 10.1101/gad.866301 PMC312644

[6] Hoogstraten D. , Bergink S., Verbiest V.H.M., Luijsterburg M.S., Geverts B., Raams A., Dinant C., Hoeijmakers J.H.J., Vermeulen W., Houtsmuller A.B. Versatile DNA damage detection by the global genome nucleotide excision repair protein XPC. J. Cell Sci. 2008; 121:2850–2859. 18682493 10.1242/jcs.031708

[7] Apelt K. , Lans H., Schärer O.D., Luijsterburg M.S. Nucleotide excision repair leaves a mark on chromatin: DNA damage detection in nucleosomes. Cell. Mol. Life Sci. 2021; 78:7925–7942. 34731255 10.1007/s00018-021-03984-7 PMC8629891

[8] Sugasawa K. Molecular mechanisms of DNA damage recognition for mammalian nucleotide excision repair. DNA Repair (Amst.). 2016; 44:110–117. 27264556 10.1016/j.dnarep.2016.05.015

[9] Xu J. , Lahiri I., Wang W., Wier A., Cianfrocco M.A., Chong J., Hare A.A., Dervan P.B., DiMaio F., Leschziner A.E. et al. Structural basis for the initiation of eukaryotic transcription-coupled DNA repair. Nature. 2017; 551:653–657. 29168508 10.1038/nature24658 PMC5907806

[10] Jia N. , Guo C., Nakazawa Y., van den Heuvel D., Luijsterburg M.S., Ogi T. Dealing with transcription-blocking DNA damage: repair mechanisms, RNA polymerase II processing and human disorders. DNA Repair (Amst.). 2021; 106:103192. 34358806 10.1016/j.dnarep.2021.103192

[11] Marteijn J.A. , Lans H., Vermeulen W., Hoeijmakers J.H.J. Understanding nucleotide excision repair and its roles in cancer and ageing. Nat. Rev. Mol. Cell Biol. 2014; 15:465–481. 24954209 10.1038/nrm3822

[12] Schärer O.D. Nucleotide excision repair in Eukaryotes. Cold Spring Harb. Perspect. Biol. 2013; 5:a012609. 24086042 10.1101/cshperspect.a012609 PMC3783044

[13] Kraemer K.H. , Patronas N.J., Schiffmann R., Brooks B.P., Tamura D., DiGiovanna J.J. Xeroderma pigmentosum, trichothiodystrophy and Cockayne syndrome: a complex genotype-phenotype relationship. Neuroscience. 2007; 145:1388–1396. 17276014 10.1016/j.neuroscience.2006.12.020 PMC2288663

[14] Lehmann A.R. , McGibbon D., Stefanini M. Xeroderma pigmentosum. Orphanet J. Rare Dis. 2011; 6:70. 22044607 10.1186/1750-1172-6-70 PMC3221642

[15] Natale V. A comprehensive description of the severity groups in Cockayne syndrome. Am. J. Med. Genet. A. 2011; 155:1081–1095. 10.1002/ajmg.a.33933 21480477

[16] Muzammal M. , Ali M.Z., Ahmad S., Huma S., Rizwan A., S. A., A.A. K., Khan M.A The molecular genetics of UV-sensitive syndrome: a rare dermal anomaly. J. Pak. Med. Assoc. 2021; 71:2391–2396. 34974577 10.47391/JPMA.03-476

[17] Natale V. , Raquer H. Xeroderma pigmentosum-Cockayne syndrome complex. Orphanet J. Rare Dis. 2017; 12:65. 28376890 10.1186/s13023-017-0616-2 PMC5379700

[18] Faghri S. , Tamura D., Kraemer K.H., DiGiovanna J.J. Trichothiodystrophy: a systematic review of 112 published cases characterises a wide spectrum of clinical manifestations. J. Med. Genet. 2008; 45:609–621. 18603627 10.1136/jmg.2008.058743 PMC3459585

[19] Lans H. , Hoeijmakers J.H.J., Vermeulen W., Marteijn J.A. The DNA damage response to transcription stress. Nat. Rev. Mol. Cell Biol. 2019; 20:766–784. 31558824 10.1038/s41580-019-0169-4

[20] Theil A.F. , Botta E., Raams A., Smith D.E.C., Mendes M.I., Caligiuri G., Giachetti S., Bione S., Carriero R., Liberi G. et al. Bi-allelic TARS Mutations Are Associated with Brittle Hair Phenotype. Am. J. Hum. Genet. 2019; 105:434–440. 31374204 10.1016/j.ajhg.2019.06.017 PMC6698936

[21] Niedernhofer L.J. , Gurkar A.U., Wang Y., Vijg J., Hoeijmakers J.H.J., Robbins P.D. Nuclear genomic instability and aging. Annu. Rev. Biochem. 2018; 87:295–322. 29925262 10.1146/annurev-biochem-062917-012239

[22] Schumacher B. , Pothof J., Vijg J., Hoeijmakers J.H.J.J. The central role of DNA damage in the ageing process. Nature. 2021; 592:695–703. 33911272 10.1038/s41586-021-03307-7 PMC9844150

[23] Cleaver J.E. Defective repair replication of DNA in xeroderma pigmentosum. Nature. 1968; 218:652–656. 5655953 10.1038/218652a0

[24] Nakazawa Y. , Yamashita S., Lehmann A.R., Ogi T. A semi-automated non-radioactive system for measuring recovery of RNA synthesis and unscheduled DNA synthesis using ethynyluracil derivatives. DNA Repair (Amst). 2010; 9:506–516. 20171149 10.1016/j.dnarep.2010.01.015

[25] Jia N. , Nakazawa Y., Guo C., Shimada M., Sethi M., Takahashi Y., Ueda H., Nagayama Y., Ogi T. A rapid, comprehensive system for assaying DNA repair activity and cytotoxic effects of DNA-damaging reagents. Nat. Protoc. 2015; 10:12–24. 25474029 10.1038/nprot.2014.194

[26] Mellon I. , Spivak G., Hanawalt P.C. Selective removal of transcription-blocking DNA damage from the transcribed strand of the mammalian DHFR gene. Cell. 1987; 51:241–249. 3664636 10.1016/0092-8674(87)90151-6

[27] Venema B. , Mullenders L.H.F., Natarajan A.T., Van Zeeland A.A., Mayne L.V. The genetic defect in cockayne syndrome is associated with a defect in repair of UV-induced DNA damage in transcriptionally active DNA. Proc. Natl. Acad. Sci. U.S.A. 1990; 87:4707–4711. 2352945 10.1073/pnas.87.12.4707 PMC54186

[28] Van Houten B. , Cheng S., Chen Y. Measuring gene-specific nucleotide excision repair in human cells using quantitative amplification of long targets from nanogram quantities of DNA. Mutat. Res. - DNA Repair. 2000; 460:81–94. 10882849 10.1016/s0921-8777(00)00018-5

[29] Guo J. , Hanawalt P.C., Spivak G. Comet-FISH with strand-specific probes reveals transcription-coupled repair of 8-oxoGuanine in human cells. Nucleic Acids Res. 2013; 41:7700–7712. 23775797 10.1093/nar/gkt524 PMC3763531

[30] Wienholz F. , Vermeulen W., Marteijn J.A. Amplification of unscheduled DNA synthesis signal enables fluorescence-based single cell quantification of transcription-coupled nucleotide excision repair. Nucleic Acids Res. 2017; 45:gkw1360. 10.1093/nar/gkw1360 PMC5436002 28088761

[31] Nakazawa Y. , Hara Y., Oka Y., Komine O., van den Heuvel D., Guo C., Daigaku Y., Isono M., He Y., Shimada M. et al. Ubiquitination of DNA Damage-Stalled RNAPII Promotes Transcription-Coupled Repair. Cell. 2020; 180:1228–1244. 32142649 10.1016/j.cell.2020.02.010

[32] Proticć-sabyić M. , Kraemer K.H. Host cell reactivation by human cells of DNA expression vectors damaged by ultraviolet radiation or by acid-heat treatment. Carcinogenesis. 1986; 7:1765–1770. 3463438 10.1093/carcin/7.10.1765

[33] Mayne L. , Lehmann A.R. Failure of RNA synthesis to recover after UV irradiation: an early defect in cells from individuals with Cockayne syndrome and xeroderma pigmentosum. Mutat. Res. 1982; 96:140. 6174225

[34] Farla P. , Hersmus R., Geverts B., Mari P.O., Nigg A.L., Dubbink H.J., Trapman J., Houtsmuller A.B. The androgen receptor ligand-binding domain stabilizes DNA binding in living cells. J. Struct. Biol. 2004; 147:50–61. 15109605 10.1016/j.jsb.2004.01.002

[35] Farla P. , Hersmus R., Trapman J., Houtsmuller A.B. Antiandrogens prevent stable DNA-binding of the androgen receptor. J. Cell Sci. 2005; 118:4187–4198. 16141232 10.1242/jcs.02546

[36] Theil A.F. , Mandemaker I.K., van den Akker E., Swagemakers S.M.A., Raams A., Wüst T., Marteijn J.A., Giltay J.C., Colombijn R.M., Moog U. et al. Trichothiodystrophy causative TFIIEβ mutation affects transcription in highly differentiated tissue. Hum. Mol. Genet. 2017; 26:4689–4698. 28973399 10.1093/hmg/ddx351 PMC5886110

[37] Van Gool A.J. , Citterio E., Rademakers S., Van Os R., Vermeulen W., Constantinou A., Egly J.M., Bootsma D., Hoeijmakers J.H.J. The Cockayne syndrome B protein, involved in transcription-coupled DNA repair, resides in an RNA polymerase II-containing complex. EMBO J. 1997; 16:5955–5965. 9312053 10.1093/emboj/16.19.5955 PMC1170226

[38] Nabet B. , Roberts J.M., Buckley D.L., Paulk J., Dastjerdi S., Yang A., Leggett A.L., Erb M.A., Lawlor M.A., Souza A. et al. The dTAG system for immediate and target-specific protein degradation. Nat. Chem. Biol. 2018; 14:431–441. 29581585 10.1038/s41589-018-0021-8 PMC6295913

[39] Campeau E. , Ruhl V.E., Rodier F., Smith C.L., Rahmberg B.L., Fuss J.O., Campisi J., Yaswen P., Cooper P.K., Kaufman P.D. A versatile viral system for expression and depletion of proteins in mammalian cells. PLoS One. 2009; 4:e6529. 19657394 10.1371/journal.pone.0006529 PMC2717805

[40] Brenner S. The genetics of caenorhabditis elegans. Genetics. 1974; 77:71–94. 4366476 10.1093/genetics/77.1.71 PMC1213120

[41] Zhang L. , Ward J.D., Cheng Z., Dernburg A.F. The auxin-inducible degradation (AID) system enables versatile conditional protein depletion in C. elegans. Dev. 2015; 142:4374–4384. 10.1242/dev.129635 PMC4689222 26552885

[42] Lans H. , Marteijn J.A., Schumacher B., Hoeijmakers J.H.J., Jansen G., Vermeulen W. Involvement of global genome repair, transcription coupled repair, and chromatin remodeling in UV DNA damage response changes during developm. PLoS Genet. 2010; 6:41. 10.1371/journal.pgen.1000941 PMC2865526 20463888

[43] Smith J.R. , Maguire S., Davis L.A., Alexander M., Yang F., Chandran S., Ffrench-Constant C., Pedersen R.A. Robust, Persistent Transgene Expression in Human Embryonic Stem Cells Is Achieved with AAVS1-Targeted Integration. Stem Cells. 2008; 26:496–504. 18024421 10.1634/stemcells.2007-0039

[44] Van Hoffen A. , Venema J., Meschini R., Van Zeeland A.A., Mullenders L.H.F. Transcription-coupled repair removes both cyclobutane pyrimidine dimers and 6-4 photoproducts with equal efficiency and in a sequential way from transcribed DNA in xeroderma pigmentosum group C fibroblasts. EMBO J. 1995; 14:360–367. 7835346 10.1002/j.1460-2075.1995.tb07010.x PMC398090

[45] Mitchell D.L. , Haipek C.A., Clarkson J.M. (6–4) Photoproducts are removed from the DNA of UV-irradiated mammalian cells more efficiently than cyclobutane pyrimidine dimers. Mutat. Res. - Mutat. Res. Lett. 1985; 143:109–112. 10.1016/s0165-7992(85)80018-x 4010689

[46] Gyenis Á. , Umlauf D., Újfaludi Z., Boros I., Ye T., Tora L. UVB Induces a Genome-Wide Acting Negative Regulatory Mechanism That Operates at the Level of Transcription Initiation in Human Cells. PLoS Genet. 2014; 10:e1004483. 25058334 10.1371/journal.pgen.1004483 PMC4109906

[47] Rockx D.A.P. , Mason R., Van Hoffen A., Barton M.C., Citterio E., Bregman D.B., Van Zeeland A.A., Vrieling H., Mullenders L.H.F. UV-induced inhibition of transcription involves repression of transcription initiation and phosphorylation of RNA polymerase II. Proc. Natl. Acad. Sci. U.S.A. 2000; 97:10503–10508. 10973477 10.1073/pnas.180169797 PMC27054

[48] Muñoz M.J. , Santangelo M.S.P., Paronetto M.P., de la Mata M., Pelisch F., Boireau S., Glover-Cutter K., Ben-Dov C., Blaustein M., Lozano J.J. et al. DNA damage regulates alternative splicing through inhibition of RNA polymerase II elongation. Cell. 2009; 137:708–720. 19450518 10.1016/j.cell.2009.03.010

[49] Tufegdžić Vidaković A. , Mitter R., Kelly G.P., Neumann M., Harreman M., Rodríguez-Martínez M., Herlihy A., Weems J.C., Boeing S., Encheva V. et al. Regulation of the RNAPII pool is integral to the DNA damage response. Cell. 2020; 180:1245–1261. 32142654 10.1016/j.cell.2020.02.009 PMC7103762

[50] Steurer B. , Janssens R.C., Geijer M.E., Aprile-Garcia F., Geverts B., Theil A.F., Hummel B., van Royen M.E., Evers B., Bernards R. et al. DNA damage-induced transcription stress triggers the genome-wide degradation of promoter-bound Pol II. Nat. Commun. 2022; 13:3624. 35750669 10.1038/s41467-022-31329-w PMC9232492

[51] Su P.-R. , You L., Beerens C., Bezstarosti K., Demmers J., Pabst M., Kanaar R., Hsu C.-C., Chien M.-P. Microscopy-based single-cell proteomic profiling reveals heterogeneity in DNA damage response dynamics. Cell Reports Methods. 2022; 2:100237. 35784653 10.1016/j.crmeth.2022.100237 PMC9243628

[52] Slyskova J. , Sabatella M., Ribeiro-Silva C., Stok C., Theil A.F., Vermeulen W., Lans H. Base and nucleotide excision repair facilitate resolution of platinum drugs-induced transcription blockage. Nucleic Acids Res. 2018; 46:9537–9549. 30137419 10.1093/nar/gky764 PMC6182164

[53] Damsma G.E. , Alt A., Brueckner F., Carell T., Cramer P. Mechanism of transcriptional stalling at cisplatin-damaged DNA. Nat. Struct. Mol. Biol. 2007; 14:1127–1133. 17994106 10.1038/nsmb1314

[54] Enoiu M. , Jiricny J., Schärer O.D. Repair of cisplatin-induced DNA interstrand crosslinks by a replication-independent pathway involving transcription-coupled repair and translesion synthesis. Nucleic Acids Res. 2012; 40:8953–8964. 22810206 10.1093/nar/gks670 PMC3467066

[55] Ang W.H. , Myint M., Lippard S.J. Transcription inhibition by platinum-DNA cross-links in live mammalian cells. J. Am. Chem. Soc. 2010; 132:7429–7435. 20443565 10.1021/ja101495v PMC2877768

[56] Alekseev S. , Ayadi M., Brino L., Egly J.M., Larsen A.K., Coin F. A small molecule screen identifies an inhibitor of DNA repair inducing the degradation of TFIIH and the Chemosensitization of tumor cells to platinum. Chem. Biol. 2014; 21:398–407. 24508195 10.1016/j.chembiol.2013.12.014

[57] Neklesa T. , Snyder L.B., Willard R.R., Vitale N., Raina K., Pizzano J., Gordon D., Bookbinder M., Macaluso J., Dong H. et al. Abstract 5236: ARV-110: An androgen receptor PROTAC degrader for prostate cancer. Cancer Res. 2018; 78:5236–5236.

[58] Cromm P.M. , Samarasinghe K.T.G., Hines J., Crews C.M. Addressing kinase-independent functions of Fak via PROTAC-mediated degradation. J. Am. Chem. Soc. 2018; 140:17019–17026. 30444612 10.1021/jacs.8b08008

[59] Lehmann A.R. Three complementation groups in Cockayne syndrome. Mutat. Res. Mol. Mech. Mutagen. 1982; 106:347–356. 10.1016/0027-5107(82)90115-4 6185841

[60] Lans H. , Vermeulen W. Tissue specific response to DNA damage: C. elegans as role model. DNA Repair (Amst). 2015; 32:141–148. 25957488 10.1016/j.dnarep.2015.04.025

[61] Lans H. , Vermeulen W. Nucleotide excision repair in *Caenorhabditis elegans*. Mol. Biol. Int. 2011; 2011:542795. 22091407 10.4061/2011/542795 PMC3195855

[62] Babu V. , Hofmann K., Schumacher B. A C. elegans homolog of the Cockayne syndrome complementation group A gene. DNA Repair (Amst). 2014; 24:57–62. 25453470 10.1016/j.dnarep.2014.09.011 PMC4255241

[63] Gorbunova V. , Seluanov A., Mao Z., Hine C. Changes in DNA repair during aging. Nucleic Acids Res. 2007; 35:7466–7474. 17913742 10.1093/nar/gkm756 PMC2190694

[64] Meyer J.N. , Boyd W.A., Azzam G.A., Haugen A.C., Freedman J.H., Van Houten B. Decline of nucleotide excision repair capacity in aging Caenorhabditis elegans. Genome Biol. 2007; 8:R70. 17472752 10.1186/gb-2007-8-5-r70 PMC1929140

[65] Gyenis A. , Chang J., Demmers J.J.P.G., Bruens S.T., Barnhoorn S., Brandt R.M.C., Baar M.P., Raseta M., Derks K.W.J., Hoeijmakers J.H.J. et al. Genome-wide RNA polymerase stalling shapes the transcriptome during aging. Nat. Genet. 2023; 55:268–279. 36658433 10.1038/s41588-022-01279-6 PMC9925383

[66] Astin J.W. , O’Neil N.J., Kuwabara P.E Nucleotide excision repair and the degradation of RNA pol II by the Caenorhabditis elegans XPA and Rsp5 orthologues, RAD-3 and WWP-1. DNA Repair (Amst). 2008; 7:267–280. 18053776 10.1016/j.dnarep.2007.10.004

[67] Sabatella M. , Theil A.F., Ribeiro-Silva C., Slyskova J., Thijssen K., Voskamp C., Lans H., Vermeulen W. Repair protein persistence at DNA lesions characterizes XPF defect with Cockayne syndrome features. Nucleic Acids Res. 2018; 46:9563–9577. 30165384 10.1093/nar/gky774 PMC6182131

[68] Dinant C. , Ampatziadis-Michailidis G., Lans H., Tresini M., Lagarou A., Grosbart M., Theil A.F., vanCappellen W.A., Kimura H., Bartek J. et al. Enhanced chromatin dynamics by fact promotes transcriptional restart after UV-induced DNA damage. Mol. Cell. 2013; 51:469–479. 23973375 10.1016/j.molcel.2013.08.007

[69] Mourgues S. , Gautier V., Lagarou A., Bordier C., Mourcet A., Slingerland J., Kaddoum L., Coin F., Vermeulen W., De Peredo A.G. et al. ELL, a novel TFIIH partner, is involved in transcription restart after DNA repair. Proc. Natl. Acad. Sci. U.S.A. 2013; 110:17927–17932. 24127601 10.1073/pnas.1305009110 PMC3816466

[70] Adam S. , Polo S.E., Almouzni G. Transcription recovery after DNA damage requires chromatin priming by the H3.3 histone chaperone HIRA. Cell. 2013; 155:94. 24074863 10.1016/j.cell.2013.08.029

[71] Oksenych V. , Zhovmer A., Ziani S., Mari P.-O., Eberova J., Nardo T., Stefanini M., Giglia-Mari G., Egly J.-M., Coin F. Histone methyltransferase DOT1L drives recovery of gene expression after a genotoxic attack. PLoS Genet. 2013; 9:e1003611. 23861670 10.1371/journal.pgen.1003611 PMC3701700

[72] Wang S. , Meyer D.H., Schumacher B. H3K4me2 regulates the recovery of protein biosynthesis and homeostasis following DNA damage. Nat. Struct. Mol. Biol. 2020; 27:1165–1177. 33046905 10.1038/s41594-020-00513-1

[73] Sacchetti A. , El Sewedy T., Nasr A.F., Alberti S. Efficient GFP mutations profoundly affect mRNA transcription and translation rates. FEBS Lett. 2001; 492:151–155. 11248254 10.1016/s0014-5793(01)02246-3

[74] Wu S. , Hu Y., Wang J.L., Chatterjee M., Shi Y., Kaufman R.J. Ultraviolet light inhibits translation through activation of the unfolded protein response kinase PERK in the lumen of the endoplasmic reticulum. J. Biol. Chem. 2002; 277:18077–18083. 11877419 10.1074/jbc.M110164200

[75] Iordanov M.S. , Pribnow D., Magun J.L., Dinh T.H., Pearson J.A., Magun B.E. Ultraviolet radiation triggers the ribotoxic stress response in mammalian cells. J. Biol. Chem. 1998; 273:15794–15803. 9624179 10.1074/jbc.273.25.15794

[76] Deng J. , Harding H.P., Raught B., Gingras A.C., Berlanga J.J., Scheuner D., Kaufman R.J., Ron D., Sonenberg N. Activation of gcn2 in uv-irradiated cells inhibits translation. Curr. Biol. 2002; 12:1279–1286. 12176355 10.1016/s0960-9822(02)01037-0

[77] Kantor G.J. , Hull D.R. An effect of ultraviolet light on RNA and protein synthesis in nondividing human diploid fibroblasts. Biophys. J. 1979; 27:359–370. 95567 10.1016/S0006-3495(79)85223-6 PMC1328594

[78] Powley I.R. , Kondrashov A., Young L.A., Dobbyn H.C., Hill K., Cannell I.G., Stoneley M., Kong Y.W., Cotes J.A., Smith G.C.M. et al. Translational reprogramming following UVB irradiation is mediated by DNA-PKcs and allows selective recruitment to the polysomes of mRNAs encoding DNA repair enzymes. Genes Dev. 2009; 23:1207–1220. 19451221 10.1101/gad.516509 PMC2685536

