# Supplementary material for: Recovery of protein synthesis to assay DNA repair activity in transcribed genes in living cells and tissues
Source: Nucleic Acids Res. 2023 Jul 31;51(18):e93. doi: 10.1093/nar/gkad642 (PMC10570043; doi:10.1093/nar/gkad642)
Supplement: gkad642_Supplemental_Files [file gkad642_supplemental_files.zip › Supplementary Table S6 legend.docx]

**Supplementary Table S6**

Data underlying each graph of the manuscript. Title of each sheet refers to the graph of which the data is shown. Each sheet also shows the number of replicate experiments (n) and number of cells tested per experiment.
