# Supplementary material for: Recovery of protein synthesis to assay DNA repair activity in transcribed genes in living cells and tissues
Source: Nucleic Acids Res. 2023 Jul 31;51(18):e93. doi: 10.1093/nar/gkad642 (PMC10570043; doi:10.1093/nar/gkad642)
Supplement: gkad642_Supplemental_Files [file gkad642_supplemental_files.zip › van der Woude2023 revised supplementary figures.pdf]

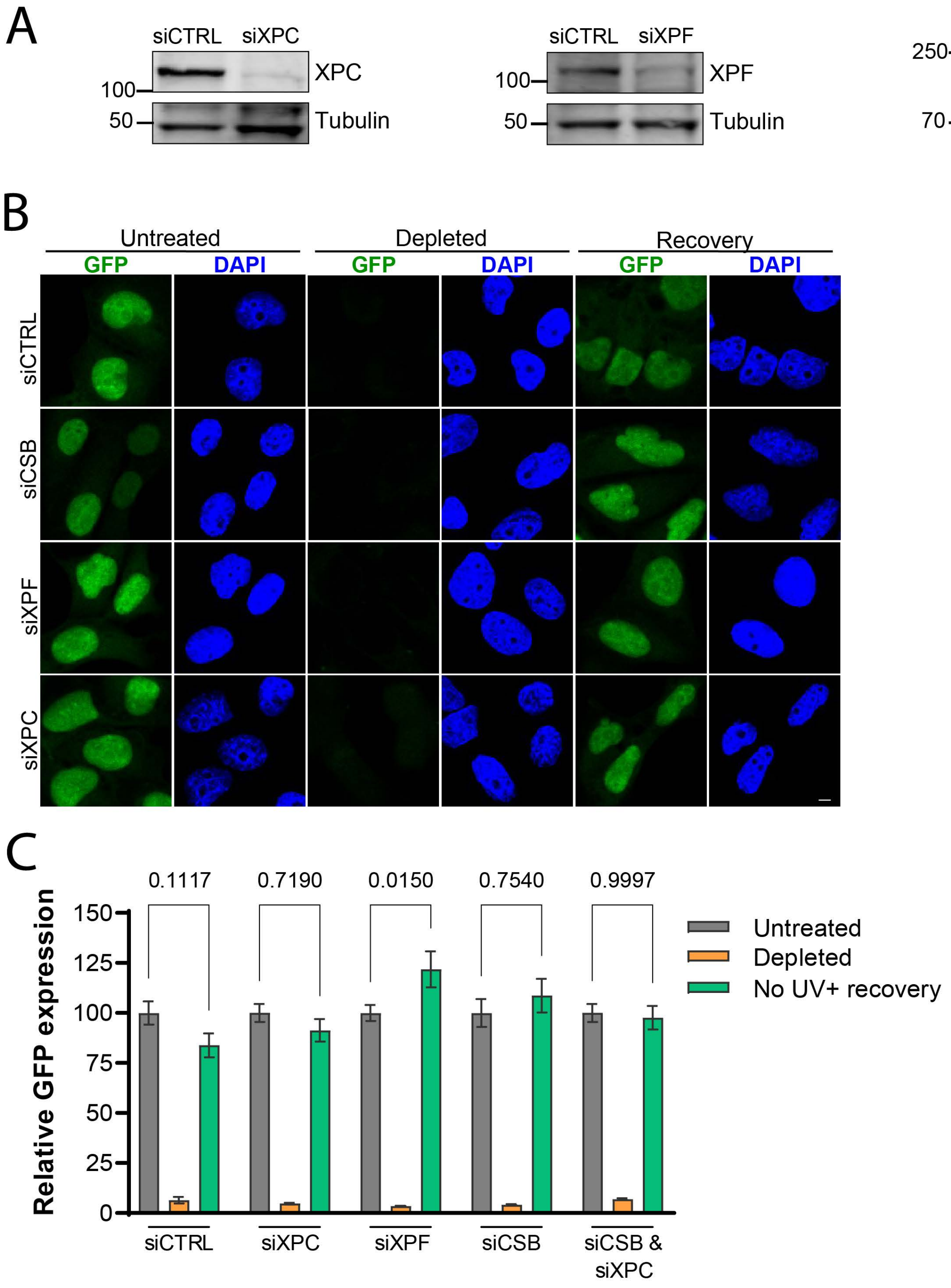

Supplementary Figure S1. Recovery of protein synthesis without UV irradiation. (**A**) Immunoblots of total U2OS cell lysates demonstrating the efficiency of siRNA-mediated protein depletion with siRNAs against XPC, XPF and CSB, probed with antibodies against the respective proteins and against tubulin or Ku70 as loading control. (**B**) Representative images of fixed EGFP-FKBPF36V-expressing U2OS cells transfected with control, CSB, XPF or XPC siRNA that were either untreated, incubated with dTAG13 for 4 h ('depleted') or incubated with dTAG13 and left to recover for 16 h ('recovery'). Scale bars 5 µm. (C) Quantification of GFP expression levels in fixed EGFP-FKBP^{F36V}-expressing U2OS cells transfected with control, XPC, XPF, CSB or CSB and XPC siRNA that were treated as shown in (B). Bars depict the mean with S.E.M. of individual cells measured in two independent experiments. The number of cells measured in respective order shown in the graph is 129, 159, 145, 136, 162, 115, 132, 173, 167, 143, 144, 130, 119, 178, and 127. Statistical differences were determined by one-way ANOVA with correction for multiple comparison. Numbers in the graph indicate p-values. Source data for the graph can be found in Supplementary Table S6.

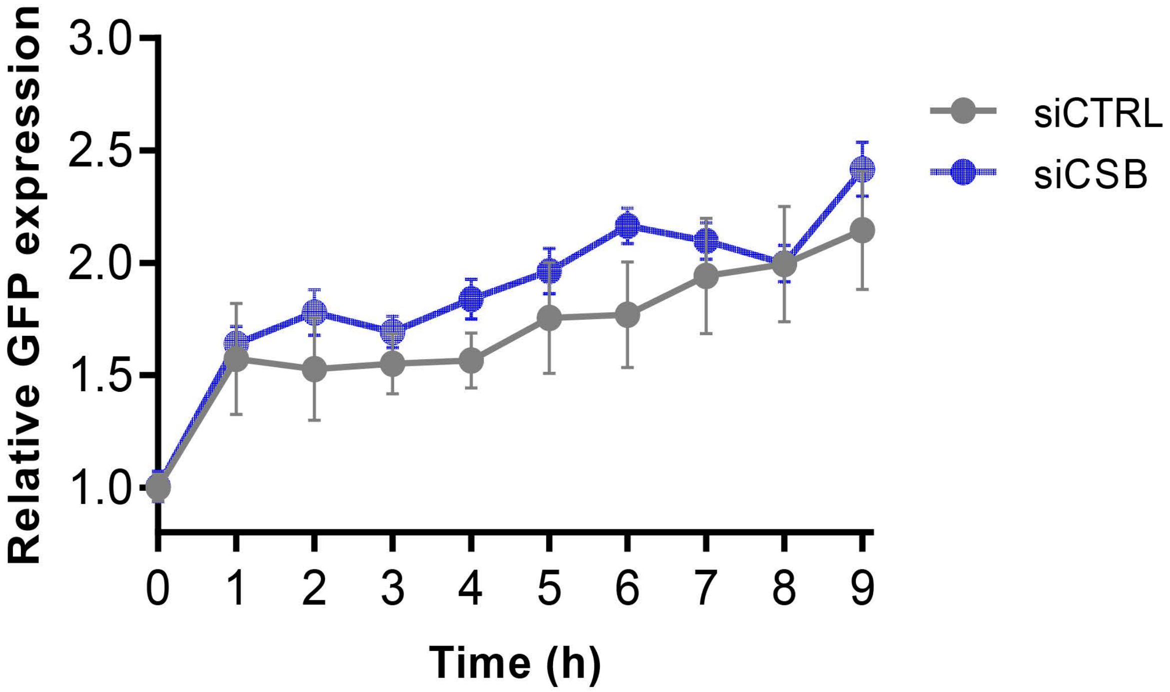

Supplementary Figure S2. Real-time recovery of protein synthesis in nonirradiated living cells. Quantification of GFP signal of EGFP-FKBP^{F36V}-expressing U2OS cells transfected with control (siCTRL) or CSB (siCSB) siRNA. Cells were incubated with dTAG13 for 8 h and imaged every hour as indicated in the graph. Depicted is the mean and S.E.M. of individual cells measured in a representative experiment. The number of measured cells in respective order of ascending time points are for siCTRL 49, 50, 53, 50, 56, 62, 64, 61, 63, and 62, and for siCSB are 59, 42, 44, 50, 50, 50, 44, 59, 46, and 47. Statistical differences were determined with unpaired t-test. Numbers in the graph indicate p-values. Source data for the graph can be found in Supplementary Table S6.

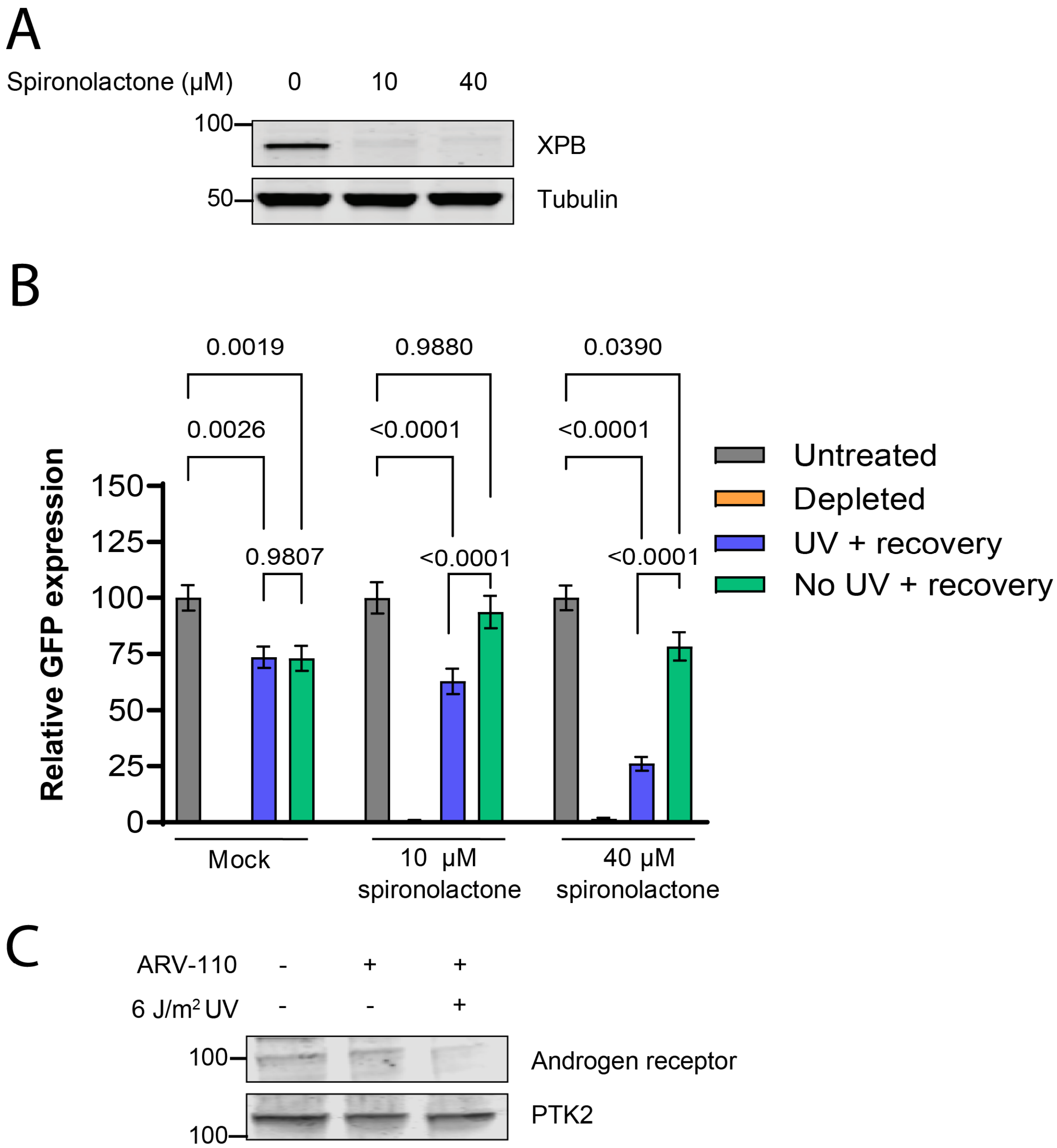

Supplementary Figure S3. Spironolactone inhibition of XPB and PTK2 expression in human fibroblasts. (**A**) Immunoblot analysis showing XPB protein levels in EGFP-FKBP^{F36V}-expressing U2OS cells treated with the indicated spironolactone concentrations for 4 h. Immunoblot is stained with antibodies against XPB and tubulin as loading control. (**B**) Quantification of GFP expression levels from mock or spironolactone-treated EGFP-FKBP^{F36V}-expressing U2OS cells that were either untreated, incubated with dTAG13 for 4 h ('depleted') or with dTAG13 for 4 h and then irradiated with 6 J/m^2 UV-C and left to recover for 16 h ('UV + recovery') or with dTAG13 for 4 h and left to recover for 16 h ('no UV + recovery'). Bars depict the mean with S.E.M. of individual cells measured in two independent experiments. The number of cells measured in respective order shown in the graph is 165, 165, 202, 140, 190, 174, 179, 241, 164, 194, 170, and 128. Statistical differences were determined by one-way ANOVA with correction for multiple comparison. Numbers in the graph indicate p-values. Source data for the graph can be found in Supplementary Table S6. (**C**) Immunoblot analysis of total cell lysate of C5RO cells either mock treated, or treated with 100 nM ARV-110 for 4 h and 6 J/m^2 UV irradiation, as indicated, using antibodies against the Androgen receptor and PTK2.

Supplementary Table S1. Experimental models: cell lines, worms, and bacterial strains

	Source	*Genetics*
U2OS – EGFP-FKBP^{F36V}	This paper	N/A
C5RO-SV40	Theil *et al.* 2017 (1)	N/A
CS3BE-SV40	GM16094; Coriell Institute	N/A
CS1AN-SV40	Van Gool *et al.* 1997 (2)	N/A
Hep3B GFP-AR	Farla *et al.* 2004 (3)	
CA1202	Zhang *et al.* 2015 (4)	*ieSi57[P(eft-3)::TIR1::mRuby] II; ieSi58 [P(eft-3)::AID::GFP] IV*
HAL526	This paper	*ieSi57 [P(eft-3)::TIR1::mRuby] II; ieSi58 [P(eft-3)::AID::GFP] IV; csb-1(ok2335)*
HAL534	This paper	*ieSi57 [P(eft-3)::TIR1::mRuby] II; ieSi58 [P(eft-3)::AID::GFP] IV; xpc-1(tm3886)*
HAL535	This paper	*ieSi57 [P(eft-3)::TIR1::mRuby] II; ieSi58 [P(eft-3)::AID::GFP] IV; csb-1(ok2335); xpc-1(tm3886)*
Escherichia coli, OP50	*Caenorhabditis* Genetics Center	WormBase ID: OP50

Supplementary Table S2. siRNA/sgRNA

si/sgRNA	*Sequence (5' to 3')*
siCTRL	UGGUUUACAUGUUGUGUGA
siCSB	GCAUGUGUCUUACGAGAUA
siXPC	GCAAAUGGCUUCUAUCGAA
siXPF	AAGACGAGCUCACGAGUAU
sgAAVS1	GGGGCCACTAGGGACAGGAT

Supplementary Table S3. Reagents

Name	*Manufacturer*	*Identifier*	*Concentration*
dTAG-13	Tocris	2064175-41-1	50 nM
Cisplatin	Sigma-Aldrich	P4394-250MG	100 µM
PROTAC FAK degrader 1	MedChemExpress	HY-119932	250 nM
R1881	Sigma-Aldrich	965-93-5	0.028 µg/ml
Spironolactone	Sigma-Aldrich	CAT#S3378	As mentioned in text
ARV-110	Selleckchem / Bioconnect	S6965	100 nM
Alt-R® S.p. Cas9 Nuclease V3	Integrated DNA technologies	1081059	According to manufacturer instruction
Lipofectamine RNAiMAX	Invitrogen	13778150	N/A
Lipofectamine™ CRISPRMAX™	Thermofisher	CMAX00-003	N/A
DAPI	Sigma	D9542	1/1000
Formaldehyde	Sigma-Aldrich	50-00-0	3.6%
Aqua-poly/mount	Polysciences Inc	CAT#18606-20	N/A
Blasticidin	InvivoGen	ant-bl-1	5 µg/ml
BSA	Sigma	A9418	5%
Triton-X100	Sigma-Aldrich	9006-19-5	0.1%
2x Laemmli sample buffer	Sigma-Aldrich	1002875919	½ dilution
NuPAGE^{TM} 4-12% Bis-Tris gels	Invitrogen	NP0321BOX	N/A
Polyvinylidene difluoride (PVDF) membrane (0.45 µm)	Milipore	IPFL00010	N/A
UV-B lamp	Philips	TL-12 tubes, 40W	N/A
UV-C lamp	Philips	254 nm; TUV lamp	N/A

Supplementary Table S4. Antibodies

Target	*Host species*	*Company*	*Identifier*	*Dilution immunoblot*
Rabbit (CF^{TM} IRDye 680)	Goat	Sigma	Sab4600200	1/10000
Mouse (CF^{TM} IRDye 770)	Goat	Sigma	Sab4600214	1/10000
Androgen receptor	Mouse	Thermo Scientific	MA5-13426	1/1000
CSB/ERCC6	Rabbit	Bethyl	A301-345A	1/1000
FAK/PTK2	Rabbit	Invitrogen	AHO0502	1/1000
GFP	Rabbit	Abcam	Ab290	1/1000
KU70	Mouse	Santa Cruz	Sc-17789	1/1000
Tubulin	Mouse	Sigma	T5168	1/10000
XPB	Rabbit	Santa Cruz	CAT#sc-293	1/1000
XPC	Rabbit	Bethyl	CAT#A301-112A	1/1000
XPF	Mouse	Santa Cruz	CAT#sc-136153	1/1000
GFP	Rabbit	Abcam	Ab290	1/1000

Supplementary Table S5. Software and algorithms

Software	*Source*
Prism Graphpad (version 9.0)	https://www.graphpad.com:443/<br>RRID:SCR_002798
ZEISS ZEN 2012 SP5 (version 14.06.201)	Carl Zeiss Microimaging Inc.
LAS AF	https://www.leica-microsystems.com/products/microscope-software/p/leica-las-x-ls/<br>RRID:SCR_013673
Fiji ImageJ	https://imagej.nih.gov/ij/<br>RRID:SCR_003070

Supplementary references

1. Theil,A.F., Mandemaker,I.K., van den Akker,E., Swagemakers,S.M.A., Raams,A., Wüst,T., Marteijn,J.A., Giltay,J.C., Colombijn,R.M., Moog,U., *et al.* (2017) Trichothiodystrophy causative TFIIEβ mutation affects transcription in highly differentiated tissue. *Hum. Mol. Genet.*, **26**, 4689–4698.

2. Van Gool,A.J., Citterio,E., Rademakers,S., Van Os,R., Vermeulen,W., Constantinou,A., Egly,J.M., Bootsma,D. and Hoeijmakers,J.H.J. (1997) The Cockayne syndrome B protein, involved in transcription-coupled DNA repair, resides in an RNA polymerase II-containing complex. *EMBO J.*, **16**, 5955–5965.

3. Farla,P., Hersmus,R., Geverts,B., Mari,P.O., Nigg,A.L., Dubbink,H.J., Trapman,J. and Houtsmuller,A.B. (2004) The androgen receptor ligand-binding domain stabilizes DNA binding in living cells. *J. Struct. Biol.*, **147**, 50–61.

4. Zhang,L., Ward,J.D., Cheng,Z. and Dernburg,A.F. (2015) The auxin-inducible degradation (AID) system enables versatile conditional protein depletion in C. elegans. *Dev.*, **142**, 4374–4384.

4. Pascal Farla, Remko Hersmus, Bart Geverts, Pierre O. Mari, Alex L. Nigg, Hendrikus J. Dubbink, Jan Trapman, Adriaan B. Houtsmuller,(2004) The androgen receptor ligand-binding domain stabilizes DNA binding in living cells. Journal of Structural Biology, **147**, 50-61

Supplementary movie S1

Movie showing recovery of GFP protein synthesis, reflecting TC-NER activity, in living U2OS cells expressing EGFP-FKBP^{F36V} and transfected with control siRNA. Cells were incubated with dTAG13 for 8 h, irradiated with 6 J/m^2 UV-C and immediately imaged every 12 min. Upper panel shows GFP fluorescence and lower panel shows bright field image. Related to Figure 2.

Supplementary movie S2

Movie showing recovery of GFP protein synthesis, reflecting TC-NER activity, in living U2OS cells expressing EGFP-FKBP^{F36V} and transfected with CSB siRNA. Cells were incubated with dTAG13 for 8 h, irradiated with 6 J/m^2 UV-C and immediately imaged every 12 min. Upper panel shows GFP fluorescence and lower panel shows bright field image. Related to Figure 2.
